# Development of second primary small‐cell lung cancer within the irradiated field after chemoradiotherapy: a report of two cases

**DOI:** 10.1002/rcr2.767

**Published:** 2021-05-25

**Authors:** Yuki Seike, Yukio Kawagishi, Akihito Bando, Ko Kimoto, Masato Hongo, Shinichi Takeda

**Affiliations:** ^1^ Department of Respiratory Medicine Kurobe City Hospital Kurobe Japan; ^2^ Department of Internal Medicine Kurobe City Hospital Kurobe Japan

**Keywords:** Chemotherapy, lung cancer, oesophageal cancer, radiation‐induced tumour, small‐cell lung carcinoma

## Abstract

Two male smokers developed small‐cell lung cancer (SCLC) as the second primary malignancy (SPM) in the irradiated field after concurrent chemoradiotherapy for locally advanced cancer, which could also be considered as a radiation‐induced tumour. A 70‐year‐old man received cisplatin and S‐1 and irradiation at 60 Gy for lung adenocarcinoma eight years previously and an 81‐year‐old man cisplatin and 5‐fluorouracil and at irradiation 60 Gy for oesophageal cancer five years previously. They sequentially received chemotherapy for SCLC, the effects of which were limited, and a refractory course was noted. Chemoradiotherapy is an effective treatment strategy for locally advanced cancer but may be relevant to the onset of SCLC as SPM.

## Graphical abstract

We present two cases of small‐cell lung cancer developed within the irradiated field relatively early after chemoradiotherapy.

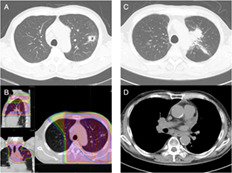

## Introduction

Advances in cancer treatment have increased overall survival and improved the cure rates. However, the number of cancer survivors who develop secondary primary malignancy (SPM) is also increasing [1]. Radiation therapy has been one of the mainstays of treatment for a variety of tumours, but it carries a risk of secondary carcinogenesis. Hallmarks of radiation‐induced tumours include a long latency period and a tendency to arise within or at the edges of prior treatment fields [1]. Advances in radiation methods and concurrent strategies in combination with chemotherapy result in improved outcomes and may increase the risk of developing radiation‐induced tumours. We present two cases of small‐cell lung cancer (SCLC) developed within the irradiated field relatively early after chemoradiotherapy.

## Case Report

### Case 1

**Figure 1:**
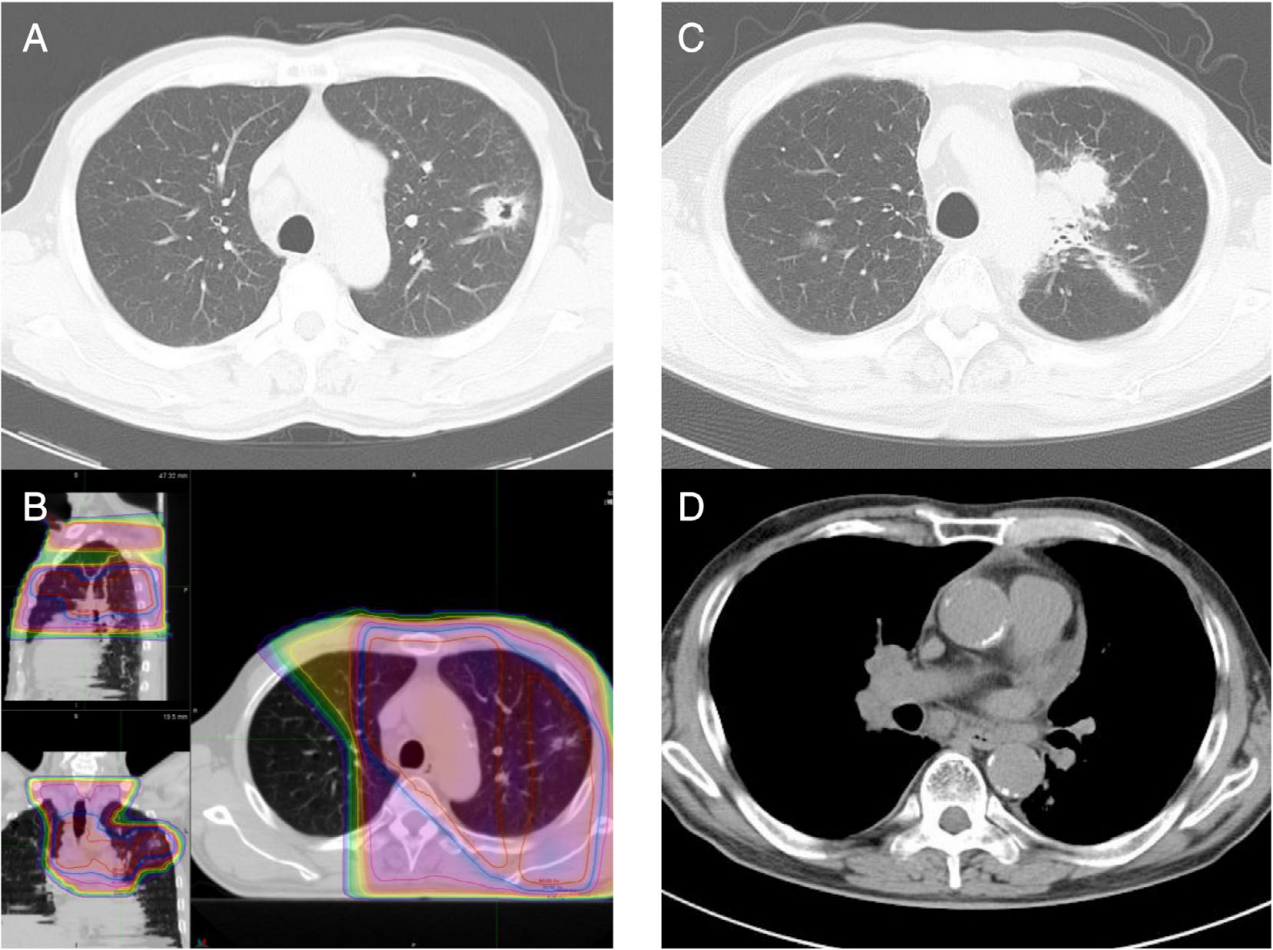
Images of case 1. (A) Thoracic computed tomography showing a pulmonary nodule with a cavity in the left upper lobe and lymph node swelling of the mediastinum at the onset of lung adenocarcinoma when the patient was 62 years. (B) Map of radiation dose distribution in radiotherapy for lung adenocarcinoma. (C) A mass seen in the left upper lobe when the patient visited the hospital for haemoptysis. (D) Swollen lymph nodes in the mediastinum and contralateral pulmonary hilum.

A 70‐year‐old male ex‐smoker (60 pack‐years) was referred to our hospital complaining of haemoptysis. At the age of 62 years, he had received concurrent chemoradiotherapy (cisplatin and S‐1, and thoracic irradiation at 60 Gy) for locally advanced lung adenocarcinoma in the left upper lobe of the lung (cT1bN3M0, stage IIIB) (Fig. 1A, B). He showed complete response with no sign of recurrence after the therapy. Eight years later, chest computed tomography (CT) revealed a tumour in the left upper lobe of the lung and lymph node swelling of the hilum and mediastinum (Fig. 1C, D). Recurrence of adenocarcinoma was suspected initially; however, pathological examination revealed small‐cell carcinoma, which was confirmed histologically and immunohistochemically (synaptophysin+, CD56+). The tumour had developed in the previous irradiated field and was therefore considered a radiation‐induced tumour. Pro‐gastrin‐releasing peptide was remarkably higher (2634.9 pg/mL) compared to 27.6 pg/mL when the first primary lung cancer developed eight years ago. He was diagnosed with SCLC (cT1bN3M0, stage IIIA). In spite of limited disease, additional thoracic irradiation was avoided because of overlap with the previous irradiation field. Combination chemotherapy with carboplatin (CBDCA), etoposide (VP‐16), and atezolizumab was started. Although the tumour shrunk temporarily, progressive disease (PD) was revealed after three courses. Amrubicin (AMR) was started and continued for five courses. Four courses of combination chemotherapy with CBDCA and irinotecan (CPT‐11) were followed. Despite a refractory course, he was treated with two additional regimens, but eventually died 13 months after onset.

### Case 2

**Figure 2:**
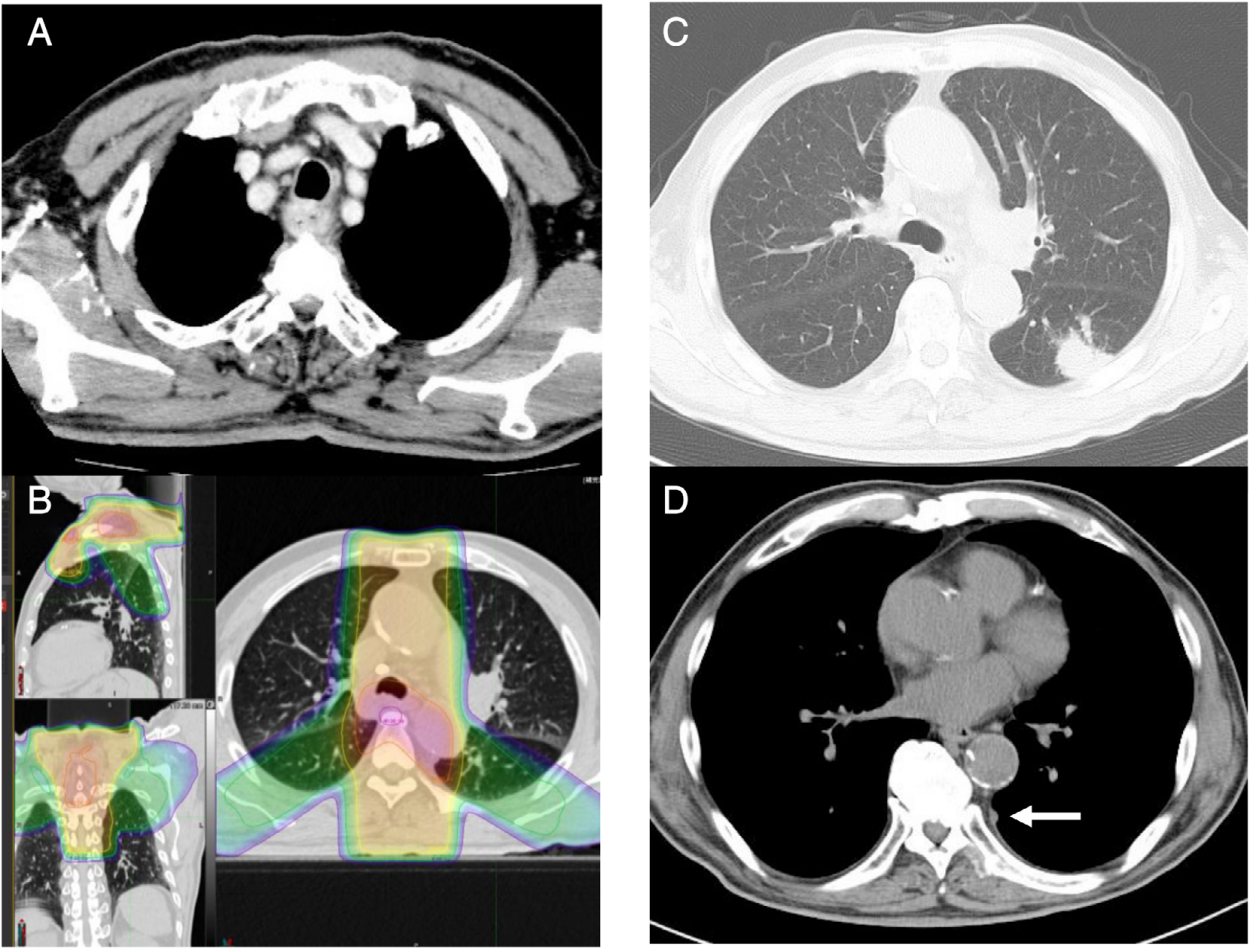
Images of case 2. (A) Thoracic computed tomography showing swelling of the oesophagus at the onset of oesophageal squamous cell carcinoma when the patient was 77 years. (B) Map of radiation dose distribution in radiotherapy for oesophageal squamous cell carcinoma. (C) A nodule seen in the left lower lobe. (D) The arrow indicates a small nodule on the pleura.

An 81‐year‐old male smoker (66 pack‐years) developed a nodule in the lower lobe of the lung on follow‐up CT after the treatment of oesophageal cancer (Fig. 2C). At the age of 77, he underwent current chemoradiotherapy (cisplatin and 5‐fluorouracil, and thoracic irradiation at 60 Gy) for locally advanced oesophageal squamous cell carcinoma (cT4aN2M0, stage IVA) (Fig. 2A). He had a complete response, and no recurrence occurred after the therapy. Five years later, a pulmonary nodule developed in the previous irradiated field (Fig. 2B). Echo‐guided needle biopsy revealed small‐cell carcinoma, which was confirmed histologically and immunohistochemically (synaptophysin+, CD56+). Another small nodule on the left pleura was positive on positron emission tomography, and he was diagnosed with extended disease SCLC with a disseminated pleural lesion (cT1cN0M1a, stage IVA) (Fig. 2D). Combination first‐line chemotherapy with CBDCA, VP‐16, and atezolizumab was initiated. The therapy yielded a marginal effect, and PD was obvious after three courses. Although AMR was tried as a second‐line therapy, no effect was obtained. Combination with CBDCA and CPT‐11 was followed yielding no effect. He eventually died six months after the onset of SCLC.

## Discussion

The onset of SPM is assumed to be relevant to a variety of factors such as genetics, ageing, smoking, and treatment, including radiotherapy and chemotherapy [1]. Concurrent chemoradiotherapy for locally advanced cancer of the lung and oesophagus has been established as a standard treatment. Platinum‐based agents and thoracic radiation are DNA‐target therapies that can be expected to have synergistic effects on tumours. The treatment can also derive cancer from DNA damage, which may make it easy for pre‐existing aberrant cells to progress to SPM. The rates of SPM are reportedly 1.8 and 2.9 per 100 patient‐years in locally advanced lung cancer treated with concurrent chemoradiotherapy [2]. In locally advanced oesophageal cancer treated with concurrent chemoradiotherapy, the rate is reportedly 19.3% over a five‐year cumulative period [3].

We encountered two cases of SCLC with SPM that developed in the irradiated field after chemoradiotherapy. In our review of the literature, there are six patients who developed second primary lung cancer within the irradiated field after chemoradiotherapy; our two cases are addition to these cases (Table 1) [2, 4, 5, 6, 7, 8]. All patients had a smoking history and received platinum‐based chemotherapy. The duration between the onset of first primary cancer and SPM was 1.8–8.7 years, which was shorter than the average onset time of radiation‐induced tumours. In six of seven cases, excluding one with SCLC as the first cancer, the histology of the SPM was small‐cell carcinoma. This could be a coincidence, and small‐cell carcinoma is more likely to be recognized as SPM because non‐SCLC was the most common first cancer. However, some cases of SCLC after chemoradiotherapy have been reported to recur within the irradiated field after a long disease‐free time of more than a decade [9]. These patients may have developed secondary primary SCLC rather than recurrence. The risk of SCLC onset reportedly increases in patients with Hodgkin's lymphoma treated with radiotherapy, high‐dose atomic bomb survivors, and uranium miners [10].

**Table 1:** Second primary LC within the irradiated field after chemoradiotherapy.

Author (reference)	Age (years)	Gender	Smoking (pack‐year)	First cancer	Chemotherapy	Dose of radiation	Duration (years)	Second cancer	Treatment	Prognosis (months)
Kawaguchi, 2006 [4]	66	M	+	LC (Ad, Sq)	Platinum doublet	ND	7.9	LC (Undiff)	ND	ND
Matsui, 2010 [5]	77	F	50	LC (Sq)	CBDCA/PTX	63.6	5	SCLC	CBDCA/CPT‐11/radiation	9, dead
Tanaka, 2015 [6]	67	M	120	LC (Sq)	CDDP/MMC/VDS	60	8	SCLC	CDDP/CPT‐11	12, alive
Takano, 2016 [7]	81	M	53	Mediastinal cancer	CBDCA/PTX	60	3	SCLC	CBDCA/VP16	4, dead
Makimoto, 2018 [2]	68	M	+	NSCLC	CDDP/DTX	46	8.7	SCLC	Chemotherapy	14, dead
Inoue, 2019 [8]	57	M	37	SCLC	CDDP/VP16	36	1.8	LC (Ad)	Surgery/UFT	ND
Case 1, 2021	70	M	60	LC (Ad)	CDDP/S‐1	60	8	SCLC	CBDCA/VP16/Atezo	13, dead
Case 2, 2021	81	M	66	Oesophageal cancer	CDDP/5‐FU	60	5	SCLC	CBDCA/VP16/Atezo	6, dead

5‐FU, 5‐fluorouracil; Ad, adenocarcinoma; age at onset of second primary malignancy; Atezo, atezolizumab; CBDCA, carboplatin; CDDP, cisplatin; CPT‐11, irinotecan; DTX, docetaxel; LC, lung cancer; MMC, mitomycin C; ND, no data; NSCLC, non‐SCLC; PTX, paclitaxel; S1, tegafur/gimeracil/oteracil potassium; SCLC, small‐cell lung cancer; Sq, squamous cell carcinoma; UFT, tegafur/uracil; Undifff, undifferentiated; VDS, vindesine; VP16, etoposide.

The major limitation to this report is that the onset in the irradiated field may be accidental. Secondary primary lung cancer may have developed unrelated to radiotherapy because the patients already had sufficient risk factors for lung cancer, and the time to onset may be too short to be considered a radiation‐induced tumour.

In conclusion, we report two cases of SCLC that developed in the irradiated field after concurrent chemoradiotherapy for locally advanced cancer of the lung and oesophagus. As the number of long‐term survivors increases, a long‐term treatment strategy that considers the risk of SPM is likely to be necessary.

### Disclosure Statement

Appropriate written informed consent was obtained for publication of this case report and accompanying images.

### Author Contribution Statement

Yuki Seike wrote the manuscript. Akihito Bando and Yukio Kawagishi were involved in data interpretation. Ko Kimoto and Masato Hongo were involved in data analysis. Yukio Kawagishi and Shinichi Takeda reviewed and edited the manuscript.
